# Regenerative and Anti-Senescence Potential of Extracts from Different Parts of Black Persimmon in an In Vitro Model of Vascular Endothelium

**DOI:** 10.3390/foods13213366

**Published:** 2024-10-23

**Authors:** Graziella Serio, Sina Naserian, Sawssen Ben Fraj, Georges Uzan, Carla Gentile

**Affiliations:** 1 Department of Biological, Chemical and Pharmaceutical Sciences and Technologies, University of Palermo, Viale delle Scienze, 90128 Palermo, Italy; graziella.serio01@unipa.it; 2 Institut National de la Santé Et de la Recherche Médicale (INSERM) UMR-S-MD 1197, Hôpital Paul Brousse, 94800 Villejuif, France; sina.naserian@inserm.fr (S.N.); sawssen_ben_fraj@yahoo.com (S.B.F.)

**Keywords:** bioactive compounds, antioxidants, endothelial progenitor cells, cellular senescence, *Diospyros digyna* Jacq.

## Abstract

Antioxidants are essential for mitigating oxidative stress and maintaining vascular health. Endothelial colony-forming cells (ECFCs) are pivotal in endothelial regeneration and angiogenesis and serve as a model to study the diversity of endothelial cells across various organs. This study evaluated the effects of peel, pulp, and seed extracts from *Diospyros digyna* Jacq. fruit (black persimmon) on human cord blood-derived ECFCs (CB-ECFCs) to determine how the distinct antioxidant profiles of the fruit’s different parts influence cellular functions. The extracts did not affect endothelial marker expression, cell proliferation, or nitric oxide production, indicating no cytotoxic or inflammatory effects. However, functional assays revealed that the seed extract significantly enhanced tube formation, increasing closed tubular networks by 1.5-fold. All extracts promoted cell migration, with the seed extract demonstrating the most substantial effect, surpassing even vascular endothelial growth factor (VEGF). Additionally, the seed extract exhibited the strongest reduction in cellular senescence, both before and after oxidative stress induction with H_2_O_2_. These findings underscore the potential of black persimmon extracts, especially from the seed, to enhance the regenerative capabilities of CB-ECFCs and reduce cellular senescence without affecting the normal endothelial phenotype. This positions them as promising candidates for developing endothelial cell therapies and advancing vascular regeneration.

## 1. Introduction

*Diospyros digyna* Jacq. is a tropical fruit tree native to Mexico. While it remains relatively unknown in Europe, successful cultivation experiments have been conducted in the Mediterranean area, including Sicily. *D. digyna* produces fruits with chocolate-colored pulp, often referred to as “chocolate pudding fruits” or black persimmons. The fruit is a rich source of bioactive compounds that are uniquely distributed across its different parts resulting in specific profiles of bioactivity, as we reported in a previous study [1].

In particular, we demonstrated that hydroalcoholic extracts from the fruit’s peel, pulp, and seeds possess significant levels of organic acids and polyphenols, showcasing remarkable radical scavenging and antioxidant properties in various experimental models. Specifically, the peel is particularly abundant in flavan-3-ols and phenolic acids, while the seeds are rich in organic acids, especially fumaric and citric acids. Additionally, sinapic acid and proanthocyanidins are significant components of the fruit, with A-type proanthocyanidins—known for their greater bioactivity—being consistently more prevalent than their B-type counterparts across all parts. These varying phytochemical profiles result in notable differences in the antioxidant properties of the three extracts. For instance, while the peel extract exhibits the highest radical scavenging and cellular antioxidant activity, the seed extract, despite its overall lower antioxidant capacity, uniquely enhances catalase (CAT) expression following cell exposure to oxidative stress [1]

It has been extensively demonstrated that antioxidant molecules play a crucial role in mitigating oxidative stress-induced damage within the vascular system [2,3]. By scavenging harmful reactive oxygen species (ROS), antioxidants serve as potent defenders of vascular integrity, effectively preventing oxidative damage to endothelial cells, smooth muscle cells, and other structural components of blood vessels [2]. Furthermore, their multifaceted mechanisms of action extend beyond mere scavenging activities, encompassing the modulation of signaling pathways involved in inflammation and angiogenesis [4,5,6]. Based on these outcomes, therapeutic applications of antioxidant treatments are considered a promising strategy for protecting and restoring normal vascular function following exposure to various stressors [4,7].

Endothelial cells (ECs) constitute the major active component of the vascular system. Their dynamic nature enables them to actively modulate vascular tone, permeability, and the inflammatory response, thereby exerting a profound influence over cardiovascular health [8]. Endothelial colony-forming cells (ECFCs) have emerged as a valuable model for studying endothelial biology. Unlike other models of endothelial cells obtained from large vessels or immortalized cell lines, ECFCs, as the direct progeny of endothelial progenitor cells (EPCs), exhibit the expression of the main endothelial markers, while still possessing characteristics of stem/progenitor cells, including high clonogenicity and proliferation rate [9]. Additionally, ECFCs, via differentiation into mature endothelial cells (ECs), are important in endothelial regeneration and angiogenesis, representing a valid model suitable for studying the effects of various molecules on the endothelium [10,11]. Although ECFCs can be obtained from both cord blood (CB) and adult peripheral blood (APB), those derived from CB (CB-ECFCs) demonstrate elevated stability in culture across multiple passages. Unlike APB-ECFCs, they have not specialized into tissue-specific endothelial phenotypes, making them a representative cellular model of the heterogeneity observed among ECs originating from different organs [12]. These unique characteristics and properties hold substantial promise for both basic research and clinical applications in vascular biology and regenerative medicine [12].

Despite numerous studies documenting the action of plant extracts rich in antioxidants on the function of mature endothelial cells, few have evaluated the effects of pure natural antioxidants or in the context of extracts on ECFC functions. Specifically, it has been demonstrated that plant extracts rich in polyphenols positively influence the redox balance, vitality, and functions of APB-ECFC, delaying the onset of the senescent phenotype [13,14,15]. Building upon this existing knowledge, the objective of this research was to explore the potential impact of hydroalcoholic extracts of black persimmons on endothelial regeneration, evaluating their effects on a range of functions displayed by ex vivo expanded CB-ECFCs. Through this research, we aim to deepen the complex relationship between oxidative stress and vascular dysfunction as well as the potential use of antioxidants from natural sources, including agro-food waste, to improve vascular health.

## 2. Materials and Methods

### 2.1. Isolation and Culture of CB-ECFC

The samples of CB were obtained from the St Louis Hospital CB bank (Paris, France; authorization no. AC-2022-5325) from healthy-term neonates. The use of human samples was in accordance with the Helsinki Declaration. Before being overlaid on Pancoll (Pan biotech, Aidenbach, Germany), CB was diluted in phosphate-buffered saline (PBS) containing 2 mM ethylenediaminetetraacetic acid (EDTA). After density gradient centrifugation, which was conducted in three steps—first at 400× *g* for 30 min at room temperature (RT) without a break, followed by a second centrifugation at 300× *g* for 20 min at 4 °C with a break, and concluding with a final step at 300× *g* for 10 min at 4 °C with a break—the obtained cells were seeded into 12-multiwell plates pre-coated with type-I rat-tail collagen (Corning, Glendale, AZ, USA). The plates were coated by adding 500 µL of collagen and incubating for 30 min at 37 °C. After incubation, the collagen solution was removed, and the wells were rinsed with PBS 1X. The cells were then cultured in endothelial cell basal medium-2 (EBM2) supplemented with endothelial single quotes kit containing 5% FBS (Lonza, Basel, Switzerland) (EGM2). Twenty-four hours later, a wash with PBS 1X was performed to remove non-adherent cells. The medium was replaced daily for the first 7 days and bi-daily thereafter until ECFC appearance (7–20 days). Only early passages (3 to 8) were used in subsequent experiments.

### 2.2. Characterization of Human CB-ECFCs

The phenotypic characterization of the isolated ECFCs was confirmed by flow cytometric analysis. After trypsinization, cells were washed with PBS 1X with 3% FBS and incubated for 20 min at 4 °C with (APC) conjugated anti-endothelial growth factor receptor 2/kinase insert region receptor (VEGFR-2/KDR), (FITC) conjugated anti-CD31, and (PE) conjugated anti-CD144 (vascular endothelial [VE] cadherin) (Miltenyi, Germany) diluted 1:50. Non-stained cells were used as a negative control.

### 2.3. Plant Material

*D. digyna* fruits were picked in June 2020 from trees grown in Vivai Torre (Milazzo, Sicily, Italy; 38°190 N, 15°240 E; 20 m a.s.l.). They were collected and stored at room temperature for three days until their complete ripeness. Pulp was manually separated from seeds and peel. Immediately after separation, the obtained portions of pulp, peel, and seeds were then distinctly used for the extraction process.

### 2.4. Extract Preparation

The extracts were prepared as previously described [1]. Briefly, peel, pulp, and seeds were separately homogenized. Three weighted aliquots of each sample were extracted with an ethanol/water mixture (70:30, *v*/*v*) using a 1:10 (*w*/*v*) extraction ratio. Samples were vortexed for 5 min and sonicated for 30 min at room temperature (RT), then they were stirred on a plate shaker overnight at 4 °C in the dark, and finally centrifuged (10 min at 8000× *g*, 4 °C). The extraction protocol was repeated three times for each sample. The supernatants from the three extraction cycles were collected, filtered (Millex HV 0.45 µm, Millipore, Billerica, MA), and stored at −80 °C until further analysis. For cell treatments, each extract was diluted in culture medium in order to ensure that the concentration of ethanol remains below 0.25% (*v*/*v*).

A schematic representation of the extraction process for peel, pulp, and seed extracts from *Diospyros digyna* Jacq fruit is included in the Supplementary Materials.

### 2.5. Cell Proliferation

Dye dilution and MTS assays were employed to assess CB-ECFC proliferation. Cells were cultured in 25 cm^2^ culture flasks and trypsinized at approximately 80% confluence. Exponentially growing cells were used for experiments.

For the MTS assay, cells were seeded in 96-well plates (9 × 10^3^ cells/cm^2^) in EGM2 medium and incubated in standard conditions (37 °C, 5% CO_2_, humidified atmosphere). After 8 h of incubation, solutions of pulp, peel, and seed extracts in EGM2 at concentrations of 0.5, 5, and 50 µg fresh weight (FW)/mL cell medium were added, and the cells were incubated in standard conditions. Cells treated with EGM2 medium only served as controls. Following incubation periods of 24, 48, or 72 h, MTS reagent (Promega, Madison, WI, USA) was added, and cells were incubated for an additional 2 h. Absorbance at 630 nm, related to MTS-formazan formation, was measured using a microplate reader (Multiskan EX plate reader, Thermo Labsystem, 01620 VantaaFinlandia) and corrected for background absorbance at 490 nm.

Cell viability for control and treated cells at 24, 48, and 72 h was determined by calculating the ratio of the average optical density measurements to the average optical density readings of the 96-well plate analyzed 8 h post-seeding (t = 0).

For the dye dilution assay, cells were labeled using a CellTrace™ Cell Proliferation Kit (CFSE, Invitrogen, Waltham, MA, USA) according to the manufacturer’s instructions. Then, cells were treated with different concentrations of peel, pulp, and seed extracts (0.5, 5, and 50 µg FW/mL). Labeled and untreated cells were used as control cells. After 72 h of incubation, labeled cells were harvested and analyzed by flow cytometry. The decrease in CFSE fluorescence intensity, resulting from dilution with each cell division, indicated cell proliferation. Events were acquired on an LSR Fortessa flow cytometer (BD bioscience, Franklin Lakes, NJ, USA) and analyzed using FlowJo software v10 (FlowJo, LLC, Ashland, OR, USA).

### 2.6. Expression of Endothelial Surface Protein Markers

CB-ECFCs were seeded in 12-well plates in EGM2 medium and incubated in standard conditions (37 °C, humidified air, 5% CO_2_). At 80% confluence, cells were washed two times with PBS, and then treated with different doses of peel, pulp, and seed extracts (0.5, 5, and 50 µg FW/mL cell medium). After 24 h, cells were trypsinized, and the expression of the main endothelial surface markers was estimated by flow cytometric analysis, as described above.

### 2.7. Nitric Oxide Production

CB-ECFCs were cultured in 75 cm^2^ culture flasks in EGM2 at 90% confluence. Cells were washed with PBS and starved (0.2% fetal bovine serum) for 4 h at 37 °C in a humidified, 5% CO_2_ atmosphere. Starved cells were washed with PBS and treated for 24 h with EGM2 containing peel, pulp, and seed extracts (0.5, 5, and 50 µg FW/mL cell medium). Cells incubated with EGM2 only were used as control. After a washing step with PBS, cells were incubated for 1 h with 1 µM 4-Amino-5-Methylamino-2′,7′-Difluorofluorescein Diacetate (DAF) probe (Thermofisher, Waltham, MA, USA). At the end of incubation, cells were collected by trypsinization and resuspended in PBS containing 3% FBS. Labelling was assessed by flow cytometry (Fortessa, BD bioscience, Waltham, MA, USA). Data were analyzed in the FITC channel using FlowJo V10 software (Salem, OR, USA).

### 2.8. Tube Formation

Cells were seeded on multiwell plates coated with geltrex (Thermofisher, Waltham, MA, USA) and cultured in EGM2 in the presence of different concentrations of peel, pulp, or seed extracts. Cells cultured in EGM2 only were used as control. The images of tube formation were taken at appropriate time intervals for 48 h and analyzed with ImageJ software (version 1.53J). Tube length, number of closed structures, and branching points were measured.

### 2.9. Wound Healing

CB-ECFCs were seeded in 12-well plates in EGM2. At 90% confluence, cell monolayers were scratched using 200 µL pipette tips. After two washing steps with PBS, different concentrations of peel, pulp, and seed extracts (0.5, 5, and 50 µg FW/mL cell medium) were added. Cells incubated with EGM2 only were used as control. Images of the scratches were taken at appropriate time points between time 0 (T0) and 24 h by a Nikon D5300 (Nikon Corporation, Tokyo, Japan). ImageJ software (National Institutes of Health, Bethesda, MD, USA) was used to quantify the wound area. The results were presented as relative wound density, defined as the ratio of the area occupied within the gap to the total area of the initial gap.

### 2.10. Transwell Migration

The effects on the regenerative function of CB-ECFCs were further validated by Transwell migration assay. Cells were starved overnight in basal medium containing 0.2% FBS. Starved cells were plated (16 × 10^4^ cells/cm^2^) on the top layer of Falcon 8-micron pore cell culture inserts (Corning, Glendale, AZ, USA), placed in 24-well plates. Next, 1 mL of starvation medium containing 1 ng/mL of vascular endothelial growth factor (VEGF) or appropriate concentrations of peel, pulp, and seed extracts (0.5, 5, and 50 µg FW/mL cell medium) were added below the cell permeable membrane. After 5 h of incubation, cells adhering to the top of the filter membrane were removed with a Q-tip. The migrated cells were fixed with 4% paraformaldehyde (PFA) and stained with blue RAL 555 (RAL Diagnostics, Martillac, France). ImageJ software was used to count the number of migrated cells. The results were expressed as % of migrated cells compared to control.

### 2.11. Inflammation Markers

CB-ECFCs were cultured in EGM2 until 90% confluence, then were treated for 24 h with different concentrations of peel, pulp, and seed extracts (0.5, 5, and 50 µg FW/mL cell medium) or with TNFα (1 ng/mL and 10 ng/mL). Cells treated with EGM2 only were used as control. After the incubation time, cells were collected by trypsinization, washed with PBS containing 3% FBS and stained with a mix of antibodies: PE-anti-VCAM (CD106), Biotin-anti-ICAM (CD54), APC-anti-TIE2 (Miltenyi, Gladbach, Germany), PECY7-anti-TNFR1, and streptavidin-PE-cys5 (Thermofisher, Waltham, MA, USA). The expression of the inflammatory markers was evaluated by flow cytometry Fortessa (BD bioscience, Franklin Lakes, NJ, USA) and data were analyzed by FlowJo V10 software (FlowJo, LLC, Ashland, OR, USA).

### 2.12. Senescence

CB-ECFCs were seeded into 12-well plates (12.5 × 10^4^ cells/cm^2^) in EGM2 and cultured to 80% of confluence. After a washing step with PBS, cells were starved overnight in basal medium containing 2% of FBS. Starved cells were treated for 24 h with 5 µg FW/mL cell medium of peel, pulp, and seed extracts, before and after the induction of senescence with a treatment with 400 µM H_2_O_2_ for 4 h. Cells treated with EGM2 only and cells incubated with H_2_O_2_ without the extracts were used as negative and positive control, respectively. Senescent cells were detected using a histochemical stain assay for β-galactosidase activity at pH 6 (Senescence Cells Histochemical Staining Kit, Sigma-Aldrich, St. Louis, MO, USA), performed according to the manufacturer’s instructions. Images of stained and unstained cells were taken by a Nikon D5300 (Nikon Corporation, Tokyo, Japan) using an inverted microscope (×10). The number of labeled cells was assessed by ImageJ software (National Institutes of Health, Bethesda, MD, USA).

### 2.13. Statical Analysis

Statistical analyses were performed with Prism (GraphPad) software (version 10). Data were expressed as means ± SEM. For cytometry, the MFI was normalized with cells cultured in EGM2. An unpaired Student’s *t*-test for *p*-value generation was used. A *p*-value < 0.05 was considered statistically significant (* *p* < 0.05, ** *p* < 0.01, *** *p* < 0.001, **** *p* < 0.0001).

To create the heat map, data for each parameter were compiled into a matrix format, with rows representing the different extracts and columns representing the various markers and functions assessed (proliferation, endothelial parameter measurements, migration assays, tube formation assay, and senescence assay). Each parameter was normalized to the control group (set as 100%), and the heat map was generated to visually represent the comparative effects of the three extracts on the evaluated parameters. The color scale was adjusted to represent the magnitude of changes, with blue indicating increased value and white indicating decreased value.

## 3. Results

### 3.1. Characterization of Human CB-ECFCs at Baseline

**Figure 1:**
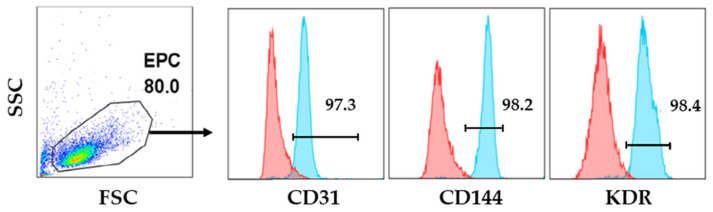
**Expression of endothelial surface markers in EPCs detected by flow cytometry.** Cells, obtained from human cord blood samples, were incubated with APC-conjugated anti-endothelial growth factor receptor 2/kinase insert region receptor (VEGFR-2/KDR), FITC-conjugated anti-CD31, and PE-conjugated anti-CD144 (vascular endothelial [VE] cadherin). Non-stained cells were used as a negative control (red peak). Similar results were obtained for at least three experiments.

It is known that ECFCs can be identified based on the expression of some EC surface markers, such as CD31, CD144, and VEGFR-2. ECFCs used in this study were isolated from human CB samples and characterized via flow cytometry after colonies’ appearance, usually between 7 and 20 days. The analysis showed that CB-ECFCs were positive for all the previously mentioned endothelial markers (Figure 1).

### 3.2. Expression of Endothelial Surface Protein Markers

**Figure 2:**
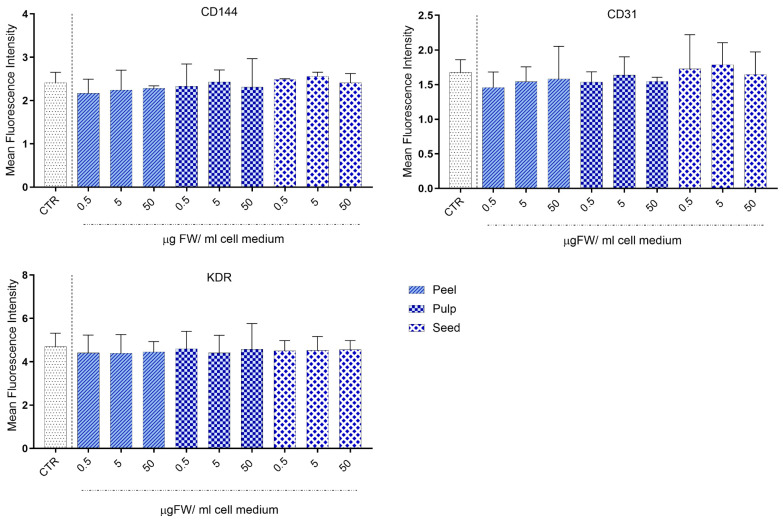
**Expression of endothelial surface markers (CD144, CD31, and KDR) in CB-ECFCs treated with black persimmon extracts.** Cells were cultured either without treatment (Control, CTR) or incubated for 24 h with varying concentrations of black persimmon peel, pulp, and seed extracts. After treatment, cells were collected and labeled with APC-conjugated anti-VEGFR-2/KDR, PE-conjugated anti-CD144, and FITC-conjugated anti-CD31 antibodies. Results are presented as the mean ± SD of three independent experiments, each conducted in triplicate. Statistical analysis was performed using one-way ANOVA followed by Tukey’s test.

The unique cell phenotype of CB-ECFCs is closely linked to their role in endothelial regeneration, angiogenesis, and the formation of new blood vessels. Therefore, it is crucial that any treatments do not disrupt the normal phenotypic profile of ECFCs. To evaluate the effect of black persimmon extracts on the expression of endothelial characteristic markers, cells were treated for 24 h with different concentrations of peel, pulp, and seed extracts (0.5, 5, and 50 µg FW/mL cell medium). Flow cytometry analysis demonstrated that the exposure of CB-ECFCs to black persimmon extracts did not induce any alterations in the expression of endothelial markers (CD144, CD31, and VGFR-2), indicating no negative effect on endothelial cell phenotype (Figure 2).

### 3.3. Impact of Black Persimmon Extracts on Cell Proliferation and NO Production in CB-ECFCs

**Figure 3:**
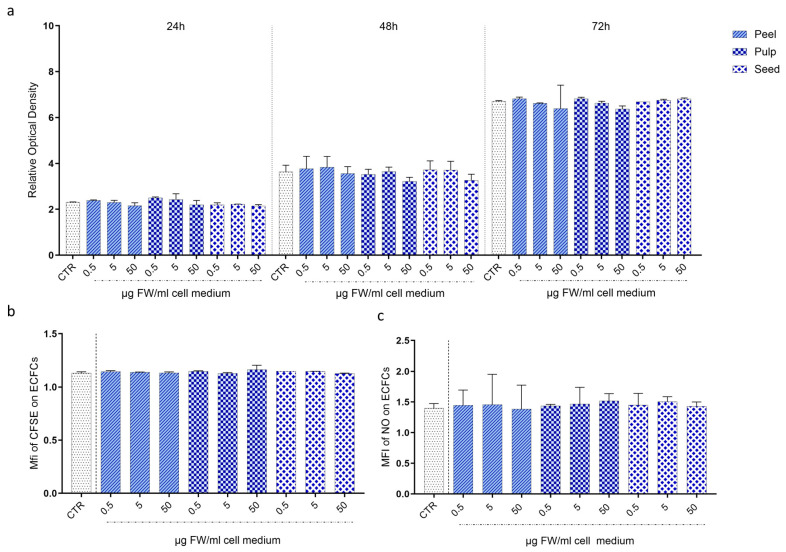
**Proliferation and NO production in CB-ECFCs treated with black persimmon extracts.** (**a**) MTS Assay: Cells were seeded in 96-well plates and incubated for 8 h, followed by treatment with peel, pulp, and seed extracts at various concentrations for 24, 48, or 72 h. Cell viability was assessed by adding MTS reagent, and absorbance at 630 nm was measured, with background correction at 490 nm. The Y-axis represents the ratio of optical density values of control and treated samples at each time point relative to the initial optical density measured at t = 0. (**b**) Dye Dilution Assay: Cells were labeled with the CellTrace™ Cell Proliferation Kit (CFSE, Invitrogen) and treated with peel, pulp, and seed extracts at different doses. Untreated labeled cells served as control. After 72 h, cells were harvested and analyzed by flow cytometry. Decreased CFSE expression indicated cell division. (**c**) NO Production: Cells were cultured to 90% confluence, starved in basal medium with 0.2% FBS for 4 h, and then treated with peel, pulp, and seed extracts for 24 h. Cells were subsequently incubated with a 1 µM DAF probe for 1 h. After incubation, cells were analyzed by flow cytometry to determine the percentage of NO-producing cells. All results are expressed as mean ± SD of three experiments carried out in triplicate. Statistical analysis was performed using one-way ANOVA followed by Tukey’s test.

The influence of black persimmon on CB-ECFC proliferation was evaluated using the MTS assay. Remarkably, the addition to the cell medium of different doses (0.5, 5, and 50 µg FW/mL of cell medium) of the three extracts did not result in any noticeable changes in cell proliferation, even after a prolonged 72 h treatment period. These findings were further corroborated by flow cytometry using tracing dye carboxyfluorescein diacetate succinimidyl ester (CellTrace™ CFSE, Invitrogen, Waltham, MA, USA). Following the appropriate incubation period, no significant differences in CFSE retention were recovered between treated and untreated cells, confirming the lack of impact on cell division. The absence of toxic effects on cells following treatments with extracts was assessed by measuring the impact on NO production, as it is well known that its reduction is closely correlated to endothelial dysfunction [16]. No significant differences in NO production were observed between treated and control cells, confirming that black persimmon extract does not have detrimental effects on CB-ECFCs (Figure 3).

### 3.4. Effect of Black Persimmon Extracts on Tube Formation by ECFCs in an Extracellular Matrix

**Figure 4:**
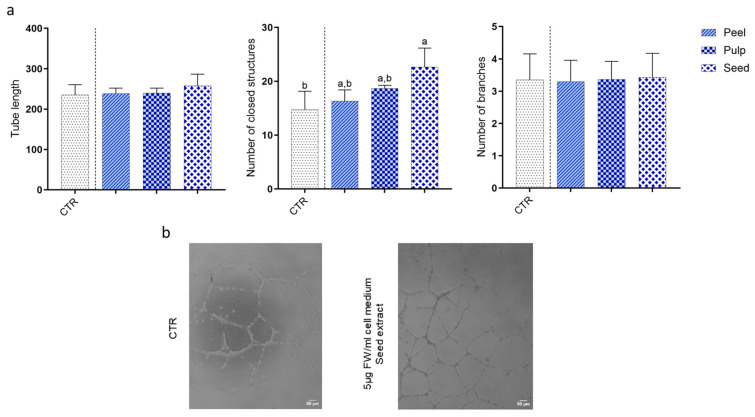
**Effect of black persimmon extracts on tube formation by CB-ECFCs.** CB-ECFC tube formation was assessed using an extracellular matrix component (Geltrex). Cells were seeded on Geltrex with or without treatment with black persimmon extracts. Cells cultured in EGM2 medium alone served as control (CTR). (**a**) Quantitative Analysis: Tube length, number of closed structures, and branch points were measured. (**b**) Representative Images: Tube formation was captured at 10× magnification over 48 h at specific time intervals. Images were analyzed using ImageJ software. Results are presented as mean ± SD of three independent experiments. Statistical significance was determined using one-way ANOVA followed by Tukey’s post hoc test. Different letters indicate statistically significant differences (*p* < 0.05), with “a” representing the highest value.

EPCs possess the remarkable ability to self-organize into tube-like structures when cultured in the presence of an extracellular matrix (ECM). To assess the impact of black persimmon extracts on the tube formation ability of ECFCs, cells were seeded on Geltrex extracellular matrix with either EGM2 alone or with EGM2 supplemented with the extracts (5 µg FW/mL medium). After seeding, cells were monitored by capturing photos at predetermined time intervals up to 48 h. While incubation with extracts from the peel and pulp of black persimmon did not affect the cells’ ability to organize into vascular-like structures, treatment with the seed extract significantly increased number of closed networks of these tubular structures without affecting the length and branches with a 1.5-fold increase in the number of closed networks, persisting up to 48 h after seeding (Figure 4).

### 3.5. Impact of Black Persimmon Extracts on the Regenerative Activity of CB-ECFCs

To evaluate the effect of black persimmon extracts on the regenerative potential of CB-ECFCs, we conducted mobility and migration assays, including the wound healing and Transwell migration assays.

In the wound healing assay, cells treated with black persimmon extracts exhibited significantly enhanced mobility compared to control cells, as evidenced by a substantial reduction in the wounded area 20 h post-scratch. Among the tested extracts, the seed extract displayed the most pronounced effect, resulting in the fastest wound closure. Specifically, after 20 h, the relative wound densities were 0.44 ± 0.08 for the control group, 0.21 ± 0.10 for the peel extract, 0.28 ± 0.10 for the pulp extract, and 0.07 ± 0.06 for the seed extract.

The positive impact of black persimmon extracts on cell migration was further corroborated by the Transwell migration assay. Treatment with black persimmon extracts significantly improved the migration of CB-ECFCs compared to untreated control, with the seed extract again demonstrating the most substantial effect. After 5 h of treatment under basal conditions, the percentage of migrated cells relative to the control group was 134.17 ± 4.24% for the peel extract, 128.92 ± 12.78% for the pulp extract, and 204.78 ± 18.29% for the seed extract.

**Figure 5:**
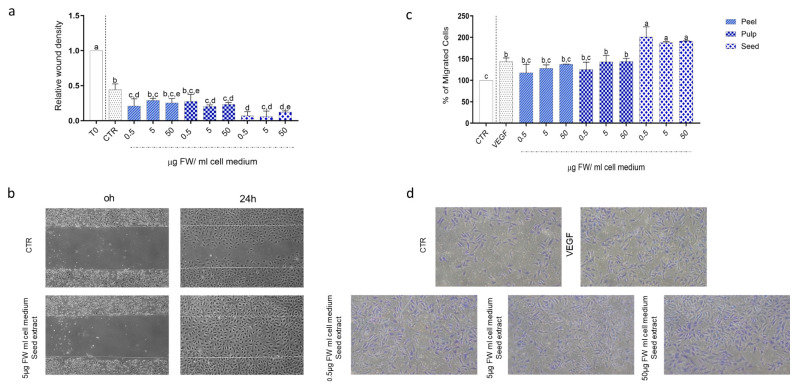
**Effect of black persimmon extracts on CB-ECFC migration assessed by wound healing and Transwell assays.** Wound Healing Assay: CB-ECFCs were cultured in EGM2 until 90% confluence, then scratched and either left untreated (CTR) or treated with different concentrations of peel, pulp, and seed extracts. (**a**) Quantitative Analysis: Wound area was quantified using ImageJ software and expressed as relative wound density. (**b**) Representative Images: Images of the wound area were captured at 0 and 24 h using an inverted microscope at 4× magnification. Transwell Migration Assay: CB-ECFCs were starved overnight in basal medium with 0.2% FBS, then detached and plated on 8-micron pore inserts. The lower chamber contained starvation medium with 1 ng/mL VEGF or various concentrations of extracts. Cells in starvation medium served as control. After 5 h, migrated cells were fixed, stained with RAL 555 blue dye, and imaged with an inverted microscope at 10× magnification. (**c**) Quantitative Analysis: The number of migrated cells was counted using ImageJ software. (**d**) Representative Images: Images of migrated cells were taken using an inverted microscope at 4× magnification. Each data point represents a measured value (*n* = 3) from three independent experiments. Results are expressed as mean ± SD. Statistical significance was determined using one-way ANOVA followed by Tukey’s test. Different letters indicate statistically significant differences (*p* < 0.05), with “a” denoting the highest value.

Given the role of vascular endothelial growth factor (VEGF) in promoting angiogenesis and endothelial cell migration, we also compared its effect with that of the black persimmon extracts. Remarkably, treatment with the seed extract elicited a stronger migratory response than VEGF, with migration percentages compared to control cells of 204.78 ± 18.29 and 143.35 ± 8.75, respectively (Figure 5).

### 3.6. Impact of Black Persimmon Extracts on the Expression of Inflammatory Markers in CB-ECFCs

To evaluate the influence of black persimmon extracts on the expression of inflammatory markers, CB-ECFCs were exposed to varying doses of the extracts for 24 h. The expression levels of key pro-inflammatory markers—vascular cell adhesion molecule (VCAM), intercellular adhesion molecule (ICAM), TIE2, and tumor necrosis factor receptor 1 (TNFR1)—were evaluated. Importantly, treatment with the peel, pulp, and seed extracts did not lead to any significant increase in the expression of these markers compared to untreated cells.

**Figure 6:**
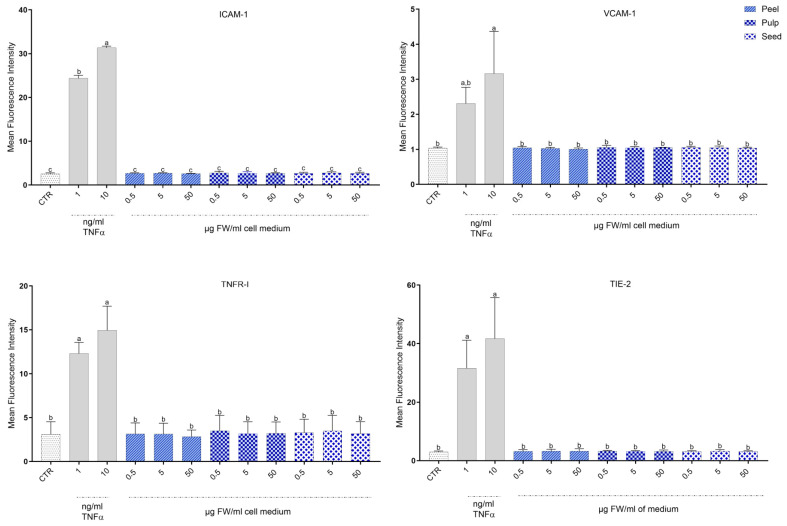
**Expression of inflammatory markers in CB-ECFCs treated with black persimmon extracts.** Cells were cultured either untreated (CTR) or incubated for 24 h with various concentrations of pulp, peel, and seed extracts, or with TNFα (1 ng/mL or 10 ng/mL). After incubation, cells were collected and stained with a mix of antibodies: Biotin-anti-ICAM1, PE-anti-VCAM1, APC-anti-TIE2, PECY7-anti-TNFR1, and streptavidin-PE-Cy5. Staining was assessed by flow cytometry and raw data were analyzed using FlowJo V10 software. Results are presented as mean ± SD of three independent experiments. Cells cultured in EGM2 without TNFα served as control (CTR). Statistical significance was determined using one-way ANOVA followed by Tukey’s test. Different letters indicate statistically significant differences (*p* < 0.05).

Additionally, the expression of inflammatory markers in the extract-treated cells, as well as in the untreated cells, remained consistently lower than that observed in cells treated with tumor necrosis factor-alpha (TNFα), which served as the positive control, both at 1 ng/mL and at 10 ng/mL (Figure 6).

### 3.7. Effect of Black Persimmon Extracts on Cellular Senescence in CB-ECFCs

Cellular senescence, marked by the irreversible cessation of cell division, is commonly triggered during extended in vitro culture. Therefore, ex vivo expansion of CB-ECFCs may gradually lead to a senescent state. To investigate the potential anti-senescent effects of black persimmon extracts, a β-galactosidase assay was performed.

The results demonstrated that all three extracts (peel, pulp, and seed) effectively reduced senescence levels induced by exposing cells to 400 μM H_2_O_2_ for 4 h. The protective effect was observed both in preventing the onset of the senescent phenotype and in restoring the normal phenotype of already senescent cells. Compared to the negative control (cells treated with culture medium only), induction of senescence with H_2_O_2_ resulted in a 2.7-fold increase in β-galactosidase-positive cells. Pre-incubation with peel, pulp, and seed extracts for 24 h significantly countered this increase, reducing the percentage of β-galactosidase-positive cells by 31.7%, 28.4%, and 57.86%, respectively, compared to the positive control (cells treated with culture medium and H_2_O_2_). Notably, the seed extract reduced the percentage of senescent cells to levels comparable to those of the negative control.

**Figure 7:**
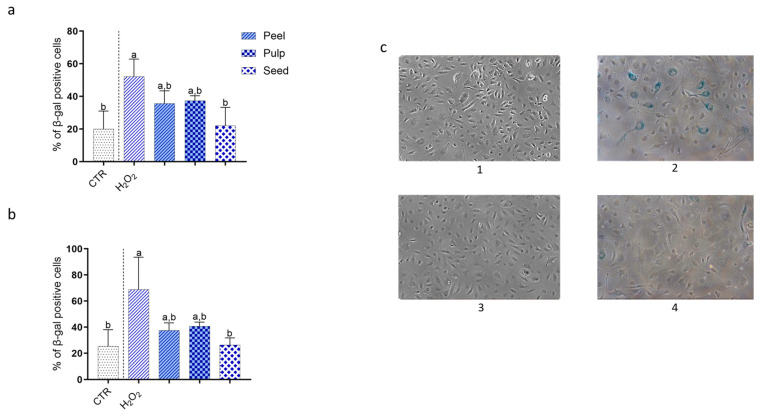
**Effects of black persimmon extracts on CB-ECFC senescence assessed by β-galactosidase staining.** Quantitative Analysis of β-Galactosidase-Positive Cells: CB-ECFCs were treated for 24 h with 5 µg FW/mL of peel, pulp, or seed extracts either before (**a**) or after (**b**) inducing senescence with 400 µM H_2_O_2_ for 4 h. Cells treated with only EGM2 served as the negative control, while cells exposed to H_2_O_2_ alone were used as the positive control. (**c**) Representative Microscopy Images: 1: Negative control; 2: Positive control; 3: Cells treated for 24 h with seed extract before senescence induction with H_2_O_2_; 4: Cells treated with seed extract after senescence induction with H_2_O_2_. Images of stained and unstained cells were captured using a Nikon D5300 (Nikon Corporation, Tokyo, Japan) with an inverted microscope at 10× magnification. Cell counts were analyzed using ImageJ software (National Institutes of Health, Bethesda, MD, USA). Results are expressed as mean ± SD from three independent experiments. Statistical significance was assessed using one-way ANOVA followed by Tukey’s test. Different letters indicate significant differences (*p* < 0.05).

Importantly, the protective activity of the extracts was also evident after senescence induction. Post-incubation with the extracts for 24 h significantly reduced the number of senescent cells previously exposed to H_2_O_2_ for 4 h. Specifically, peel, pulp, and seed extracts decreased β-galactosidase-positive cells by 45.5%, 40.77%, and 61.75%, respectively, compared to cells exposed only to H_2_O_2_. The seed extract exhibited the highest activity, restoring the number of β-galactosidase-positive cells to levels comparable to those observed in cells incubated only with culture medium (Figure 7).

### 3.8. Comparative Analysis of Extracts from Black Persimmon on Endothelial Regeneration Markers

**Figure 8:**
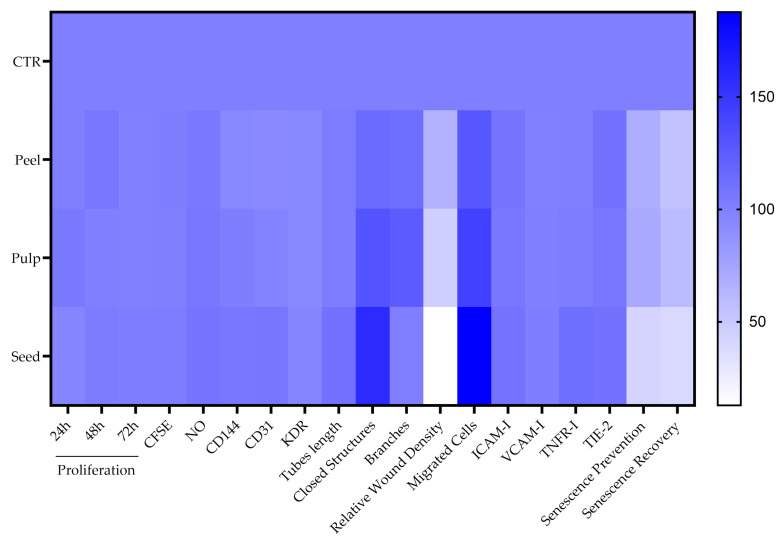
**Comparative analysis of extracts on endothelial regeneration.** The heat map was generated using GraphPad Prism (version 10). To create the heat map, data for each parameter were compiled into a matrix format, with rows representing the different extracts and columns representing the various markers and functions assessed (proliferation, endothelial parameter measurements, migration assays, tube formation assay, and senescence assay). Each parameter was normalized to the control group (set as 100%). The color scale was adjusted to represent the magnitude of changes, with blue indicating increased value and white indicating decreased value.

The heat map presented in Figure 8 provides a comprehensive analysis of the effects of three distinct extracts from black persimmon on the markers and functions associated with endothelial regeneration. A comparison of the extracts with control cells reveals that the treatments significantly impact parameters related to cell migration, vascular regeneration, and cellular senescence. In contrast, parameters associated with the non-activated endothelial phenotype and cellular proliferation show minimal change. Furthermore, the heat map offers a clear visual comparison among the three extracts, highlighting the consistent superiority of the seed extract in influencing the most responsive treatment parameters.

## 4. Discussion

Black persimmon extracts, known for their unique polyphenolic profile, have been recognized for their remarkable radical scavenging and antioxidant properties in various experimental models [1,17,18,19]. Antioxidant molecules, by mitigating oxidative stress, play a crucial role in protecting the vascular system and promoting the restoration of normal vascular function following exposure to different stressors [2]. Among the essential components of the vascular system, EPCs are involved in regeneration and re-endothelialization, contributing up to 25% to the formation of new blood vessels [20,21]. A reduction in the number and function of EPCs increases the risk of vascular diseases and impairs the restoration of a healthy vascular condition [9,22]. Moreover, it has been demonstrated that cellular senescence can limit the ability of ex vivo expanded EPCs to sustain ischemic tissue and repair [23]. Therefore, enhancing EPC functions while simultaneously preventing a senescent phenotype by pharmacological modulation during ex vivo amplification could represent a strategy for improving neovascularization after vascular diseases, including ischemia.

Considering the significance of specific EPC functions, such as mobility, migration, and network formation, as critical parameters influencing pro-angiogenic effects [24], we evaluated the impact of cell treatment with black persimmon extracts on these functions. Prior studies have reported positive effects on the migration and mobility of EPCs following treatments with both pure antioxidants, such as resveratrol and ginsenoide Rg1, as well as plant extracts, including those from Ginkgo biloba and grape skin and seeds [13,15]. In particular, cell exposure to antioxidant molecules has positive effects on the re-endothelialization ability and has prevented negative effects induced by stress conditions [13]. The findings of the present study demonstrated a notable enhancement in the motility and migration of EPCs following treatment with black persimmon extracts. Among these extracts, the seed extract exhibited the most prominent effects. Specifically, treatment with the seed extract, tested across concentrations ranging from 0.5 to 50 µg/mL in the cell medium, resulted in the most rapid healing activity compared to untreated cells or those treated with peel and pulp extracts. Of particular interest is the observed impact on cell migration after a 5-h incubation with the seed extract, which led to a twofold increase in migrating cells compared to the control condition. Moreover, the efficacy of the seed extract components in promoting cell migration surpassed that of VEGF added to the medium at a concentration of 1 ng/mL.

Several studies have emphasized the anti-inflammatory effects of antioxidant agents on endothelial cells. For instance, Huang et al. demonstrated that pretreatment with resveratrol significantly inhibited TNFα-induced mRNA expression of iNOS, ICAM-1, and IL-1β in human coronary arterial endothelial cells (HCAECs) [25]. Similarly, Liu et al. found that leonurine, an active alkaloid from traditional Chinese medicine *Herba leonuri*, exhibited cardioprotective effects, through its anti-oxidative activity, in human umbilical vein endothelial cells (HUVECs) by attenuating LPS-mediated expression and release of ICAM-1, VCAM-1, cyclooxygenase-2, and TNFα [26]. Recent studies have also shown that inflammation and oxidative stress play crucial roles in regulating EPC functions [3,27,28]. For instance, clinical studies have demonstrated that statin treatment, known for its anti-inflammatory properties, significantly enhances the mobilization of EPCs and boosts their angiogenic capabilities [29,30].

Conversely, EPCs are also highly sensitive to pro-inflammatory environments [31]. Studies have shown that the pre-treatment of EPCs with increasing doses of TNFα is directly correlated with the upregulation of endothelial inflammatory markers [11,31]. Exposure of EPCs to black persimmon extracts, compared to untreated cells, did not adversely affect cell proliferation or the expression of key endothelial surface proteins such as CD31, KDR, and CD144, nor did it alter nitric oxide (NO) production. Additionally, measuring the levels of typical endothelial inflammatory markers allowed us to exclude any proinflammatory actions of the extracts on the cells after 24 h of treatment. Specifically, while treatment of cells with 10 ng/mL of TNFα significantly increased the expression levels of ICAM-1, VCAM-1, TIE-2, and TNFR-I, exposure of the cells to the extracts for 24 h did not alter the expression of inflammatory markers compared to control cells. Despite recent evidence suggesting that a limited intensity activating stimulus can positively impact the immunomodulatory functions of EPCs, the expression levels of inflammatory markers in cells exposed to the extracts were lower compared to those treated with 1 ng/mL of TNFα. Overall, the results suggest that the treatment of EPCs with black persimmon extracts does not negatively influence the endothelial progenitor phenotype and indicate that the positive activity of the extract components on the angiogenic properties of EPCs is not attributable to an inflammatory activation.

The isolation and in vitro expansion of endothelial cells frequently lead to the gradual development of a senescent phenotype, which results in reduced proliferation rates and diminished functional capabilities. Nevertheless, this process can be mitigated by treating the cells with anti-aging agents. Extracts from *Tinospora cordifolia* leaf and *Withania somnifera* root, along with resveratrol, have been shown to delay senescence, as confirmed by β-galactosidase senescence assays in mesenchymal stem cells (MSCs), without compromising cell viability [32,33]. This capability was also observed in adult peripheral EPCs treated with Gensenoide Rg1 [14]. In this study, we examined the impact of black persimmon extracts on the prevention and treatment of cellular senescence. Our findings revealed that all three extracts were effective in reducing the senescence levels of H_2_O_2_-induced cells. Notably, the seed extract was particularly potent, as it restored the senescence levels of non-induced cells both before (prevention) and after (curing) the induction of senescence. These results suggest that the phytochemicals present in the seed extracts possess significant anti-senescent properties. Specifically, they could prevent senescence in CB-ECFCs during ex vivo expansion and counteract the aging phenotype in already senescent cells. This dual capability underscores the potential of black persimmon seed extracts as a therapeutic agent for managing cellular aging and maintaining cellular health.

## 5. Conclusions

In summary, this study highlights the promising potential of black persimmon extracts, particularly those derived from the seeds, in enhancing the functionality of CB-ECFCs. Notably, treatment with the seed extract resulted in a significant enhancement in CB-ECFCs’ mobility and migration abilities without compromising their viability and endothelial identity. Furthermore, the anti-aging activity of these extracts adds an important dimension, suggesting that black persimmons may play a crucial role in vascular health and regeneration. Collectively, these findings warrant further investigations to elucidate the precise mechanisms by which the phytochemicals found in black persimmon extracts, especially those from the seeds, enhance the function and activity of CB-ECFCs. Understanding these mechanisms could pave the way for novel therapeutic applications in regenerative medicine and anti-aging strategies.

## Data Availability

The original contributions presented in the study are included in the article/Supplementary Materials, further inquiries can be directed to the corresponding authors.

## Supplementary Materials

The following supporting information can be downloaded at: https://www.mdpi.com/article/10.3390/foods13213366/s1.
