# Supplementary figures and images for: Regenerative and Anti-Senescence Potential of Extracts from Different Parts of Black Persimmon in an In Vitro Model of Vascular Endothelium
Source: Foods. 2024 Oct 23;13(21):3366. doi: 10.3390/foods13213366 (PMC11545823; doi:10.3390/foods13213366)

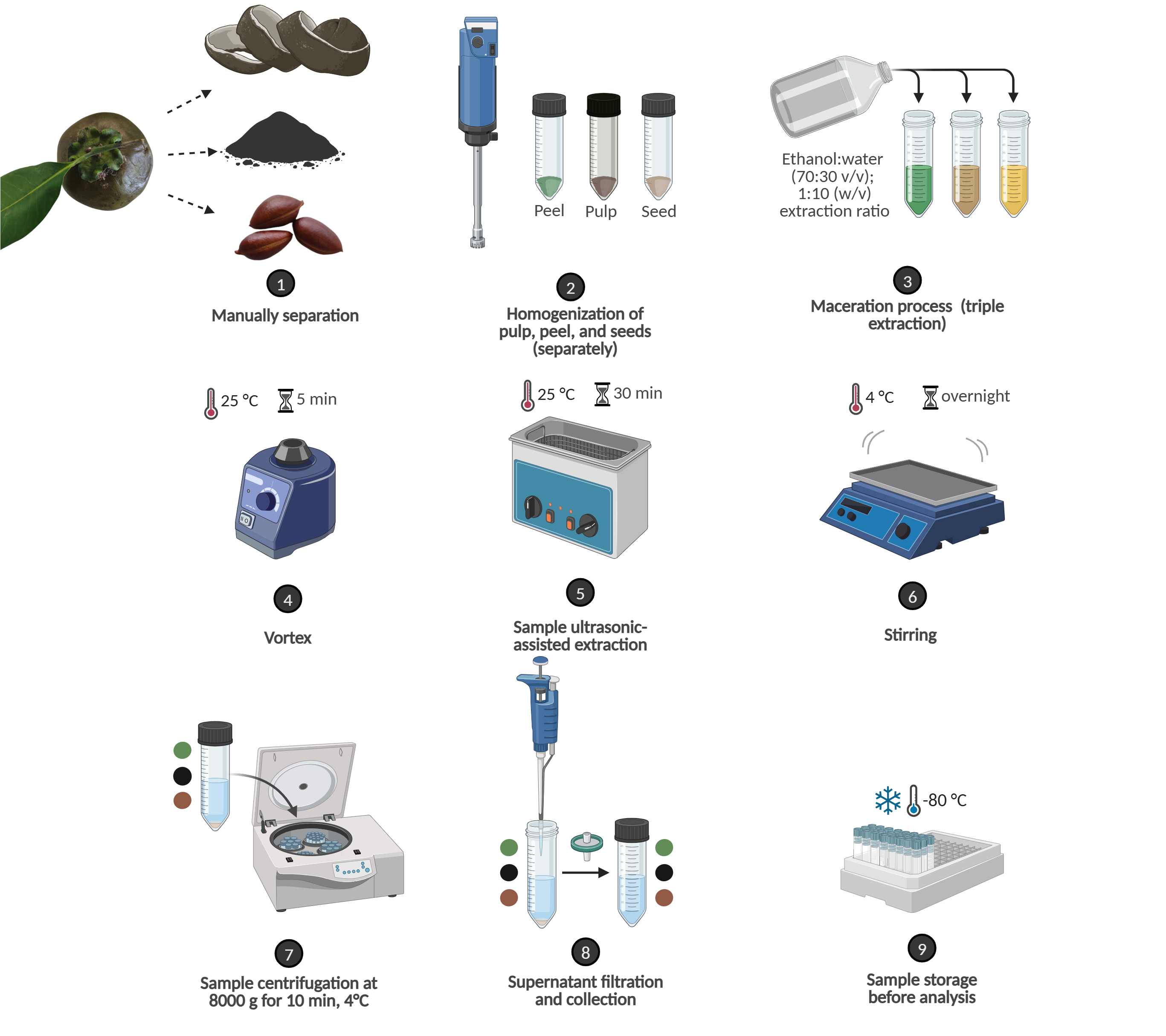

Supplement: Supplementary file 1 [file foods-13-03366-s001.zip › foods-3240561-supplementary.png]
